# Enhancing dromedary camel (*Camelus dromedarius*) healthcare: ultrasound-guided diagnostic and therapeutic interventions in the thoracic and abdominal cavities

**DOI:** 10.3389/fvets.2026.1735753

**Published:** 2026-02-04

**Authors:** Mohamed Tharwat, Hassan Barakat

**Affiliations:** 1 Department of Clinical Sciences, College of Veterinary Medicine, Qassim University, Buraidah, Saudi Arabia; 2 Department of Food Science and Human Nutrition, College of Agriculture and Food, Qassim University, Buraidah, Saudi Arabia

**Keywords:** camel healthcare, diagnostic imaging, dromedary camel, minimally invasive therapy, ultrasound-guided interventions

## Abstract

Dromedary camels (*Camelus dromedarius*) are vital to the socioeconomic and cultural fabric of arid regions, yet their healthcare is less developed than that of other domestic species. Their unique anatomy—including deep thoracoabdominal cavities, thick skin, and dense musculature—challenges conventional diagnostics and interventions. Ultrasound has emerged as a crucial, field-appropriate imaging modality, enabling rapid, non-invasive, real-time visualization of internal structures. This review examines ultrasound-guided diagnostic and therapeutic procedures in dromedary camels. Key diagnostic techniques, including thoracocentesis, abdominocentesis, portocentesis, and organ biopsies, are discussed with respect to clinical indications, protocols, advantages, and potential complications. Ultrasound guidance enhances accuracy and safety by ensuring precise needle placement, minimizing trauma, and improving diagnostic yield. Therapeutic applications, such as pleural effusion drainage and abscess evacuation, highlight ultrasound’s role in minimally invasive alternatives to surgery. Camel-specific anatomical and behavioral factors influencing ultrasonography, including sternal recumbency and adapted equipment, are addressed. Ultrasound features that differentiate types of ascitic fluid—transudates, exudates, and hemorrhagic effusions—are critical for targeted treatment. It also aids in managing complex conditions such as uroperitoneum, peritonitis, and thoracic or abdominal effusions, with ultrasound-guided paracentesis improving differentiation between urinary bladder rupture and obstruction. Challenges remain due to camel anatomy, environmental conditions, sedation risks, and the need for trained operators. Future directions include developing camel-specific protocols, portable devices, AI-assisted and tele-guided diagnostics, and integration into veterinary education. Widespread adoption of ultrasound-guided interventions can enhance camel healthcare, animal welfare, and pastoral livelihoods in arid regions worldwide.

## 1 Introduction

Dromedary camels (*Camelus dromedarius*) play a pivotal role in the socioeconomic and cultural fabric of arid and semi-arid regions, particularly in North Africa, the Middle East, and parts of Asia (1–4). As resilient animals adapted to harsh desert environments, camels contribute significantly to food security through the production of milk, meat, and fiber, and remain essential for transportation and traditional livelihoods in many pastoral communities (5, 6). Beyond their economic utility, camels also hold deep cultural and heritage value in many regions, especially in nomadic societies (7, 8).

Despite their importance, veterinary care for camels has historically lagged behind that of other large domestic species such as cattle and horses (9). This is in part due to distinct anatomical and physiological peculiarities of camels, including their large body mass, deep and compartmentalized thoracoabdominal cavities, thick and tightly adherent skin, and well-developed musculature, which collectively pose unique challenges for clinical examination and internal diagnostics (10). In addition, camels possess a unique foregut anatomy with complex stomach compartments, relatively small intercostal spaces, and a high tolerance to dehydration, all of which can alter clinical signs and complicate physical and imaging-based assessments. These characteristics limit the effectiveness of traditional diagnostic modalities such as percussion, auscultation, and manual palpation (11). Furthermore, access to specialized diagnostic facilities is often limited in remote regions where camels are primarily raised, making field-appropriate tools crucial for timely and accurate diagnosis (12).

In recent years, ultrasonography has emerged as a transformative tool in camel medicine (13). Portable ultrasound units, when applied skillfully, provide rapid, non-invasive, and real-time imaging of internal structures (14). Ultrasonography not only enhances the clinician’s ability to diagnose a wide range of thoracic and abdominal disorders, but it also allows for precise guidance of therapeutic procedures such as fluid aspiration and tissue biopsy (15–23). The technique is particularly valuable in camels, where conventional imaging techniques such as radiography are less practical due to size constraints and the need for transportation to clinical facilities (24).

In large animals, ultrasound-guided interventions are increasingly used to improve the accuracy and safety of diagnostic and therapeutic procedures by allowing real-time visualization of soft tissues and needle placement. In horses, ultrasound guidance is well established for intra-articular injections, tendon and ligament treatments, regional anesthesia, and biopsies, significantly reducing complications associated with blind techniques (25, 26). In cattle, ultrasound-guided techniques are applied for aspiration of abscesses, biopsies, fluid drainage, and reproductive interventions, extending the use of ultrasonography beyond diagnosis into minimally invasive clinical management (27). Overall, ultrasound-guided interventions enhance precision, reduce tissue trauma, and support evidence-based decision-making in large animal practice.

In dromedaries, ultrasound-guided interventions, including thoracocentesis, abdominocentesis, organ biopsies, and abscess drainages, are now being increasingly recognized for their role in improving camel health outcomes (28). These procedures, performed under real-time imaging, minimize the risk of complications while maximizing diagnostic and therapeutic success (29). Notably, camels are generally examined in a sternal recumbency position during ultrasonographic assessments, a factor that enhances stability and operator control during guided interventions (30).

The objective of this narrative review is to summarize ultrasound-guided diagnostic and therapeutic procedures in dromedary camels. The focus is specifically on practical, field- and clinic-applicable interventions, including thoracocentesis, abdominocentesis, organ biopsies, and abscess drainages, rather than on general diagnostic ultrasonography. Emphasis is placed on clinical indications, procedural techniques, diagnostic yield, and potential complications. By synthesizing recent advancements and field practices, this review aims to provide veterinary practitioners and researchers with evidence-based guidance to improve camel healthcare in both clinical and field settings.

The literature included in this review was identified through a comprehensive search of major scientific databases, including PubMed, Scopus, Web of Science, and Google Scholar. Keywords such as *dromedary camel*, *Camelus dromedarius*, *ultrasonography*, *ultrasound-guided intervention*, *thoracic diseases*, *abdominal diseases*, and *interventional procedures* were used in various combinations. Peer-reviewed articles, clinical studies, case reports, and review papers published primarily in English were considered, with particular emphasis on publications addressing practical clinical applications of ultrasonography in camels. Relevant references cited within selected articles were also screened to ensure comprehensive coverage of the topic.

## 2 Unique considerations in camel ultrasonography

Ultrasonography in dromedary camels presents distinct challenges and requirements that differ markedly from other domestic large animals (28). These differences are largely attributable to the camel’s unique anatomical and physiological characteristics, behavior during examination, and environmental context in which imaging is typically performed (13). A nuanced understanding of these factors is essential to optimize diagnostic yield and minimize procedural complications during ultrasound-guided interventions (28).

### 2.1 Camel anatomy and physiology

Dromedary camels possess a deep thoracic cavity and voluminous abdominal space, which complicates imaging of deeper structures (31). Their relatively thick, inelastic skin and the presence of dense subcutaneous fat pads, particularly in well-nourished or hydrated animals, can attenuate ultrasound waves and reduce image clarity (32). Moreover, the expansive peritoneal cavity, although advantageous for visualizing free fluid accumulations, often poses a challenge for precise localization of abdominal organs, especially in the presence of gastrointestinal gas or peritoneal fat (63). These anatomical traits necessitate tailored approaches to scanning technique and probe handling.

### 2.2 Standard examination position

A notable behavioral and practical consideration in camel ultrasonography is the positioning of the animal. Camels are almost universally examined in a state of sternal recumbency—colloquially position—due to their large size, strong musculoskeletal support, and generally calm disposition when restrained in this posture (33). This position provides a stable and safe configuration for both animal and operator, facilitating access to both ventral and lateral aspects of the thoracoabdominal region (34). However, it also limits access to dorsal anatomical structures unless the animal is carefully rolled or restrained in lateral recumbency, which is less common in field conditions (28).

### 2.3 Equipment and probe selection

Given the variability in tissue depth across anatomical regions in dromedary camels, careful selection of ultrasound probes is essential. Convex transducers with frequencies ranging from 3.5 to 5.0 MHz are commonly employed for general abdominal and thoracic assessments due to their capacity for deeper tissue penetration (28). In contrast, linear transducers operating at higher frequencies (5.0–7.5 MHz) are preferred for transrectal examination of the urogenital organs (14). In field settings, lightweight, portable ultrasound machines are increasingly utilized, offering sufficient resolution for a wide range of diagnostic applications while ensuring operator mobility and ease of deployment. For large-animal practice, considerations such as extended battery life and rugged construction are particularly important to maintain performance under harsh environmental conditions and during prolonged field use (33, 34).

### 2.4 Image optimization challenges

Several factors compromise image acquisition and interpretation in camel ultrasonography. The dense hair coat, particularly during colder seasons, can trap air and reduce probe-skin contact, leading to reverberation and shadowing artifacts (28). In addition, the camel’s robust abdominal musculature and the frequent presence of gastrointestinal gas interfere with the transmission of ultrasound waves, especially in regions such as the forestomachs and large intestines (35). To mitigate these limitations, careful site preparation—including clipping, degreasing, and ample use of acoustic gel—is essential. Furthermore, systematic scanning protocols and anatomical landmarking adapted to camel morphology are necessary to improve consistency and diagnostic accuracy (13).

## 3 Ultrasound-guided diagnostic procedures

In humans as well as in veterinary medicine, ultrasound has emerged as a cornerstone in the diagnostic landscape due to its real-time imaging capabilities, non-invasive nature, and ability to guide precise sampling from fluid-filled or solid structures (36). In dromedary camels, whose anatomy and temperament often complicate traditional diagnostic techniques, ultrasound-guided interventions provide enhanced accuracy, improved safety, and greater diagnostic yield (28). In camels, these advantages are particularly important because of the species’ large size and the logistical challenges of referral imaging. The following section outlines key ultrasound-guided diagnostic procedures with an emphasis on their clinical utility in dromedary camel medicine.

### 3.1 Thoracocentesis

**Figure 1:**
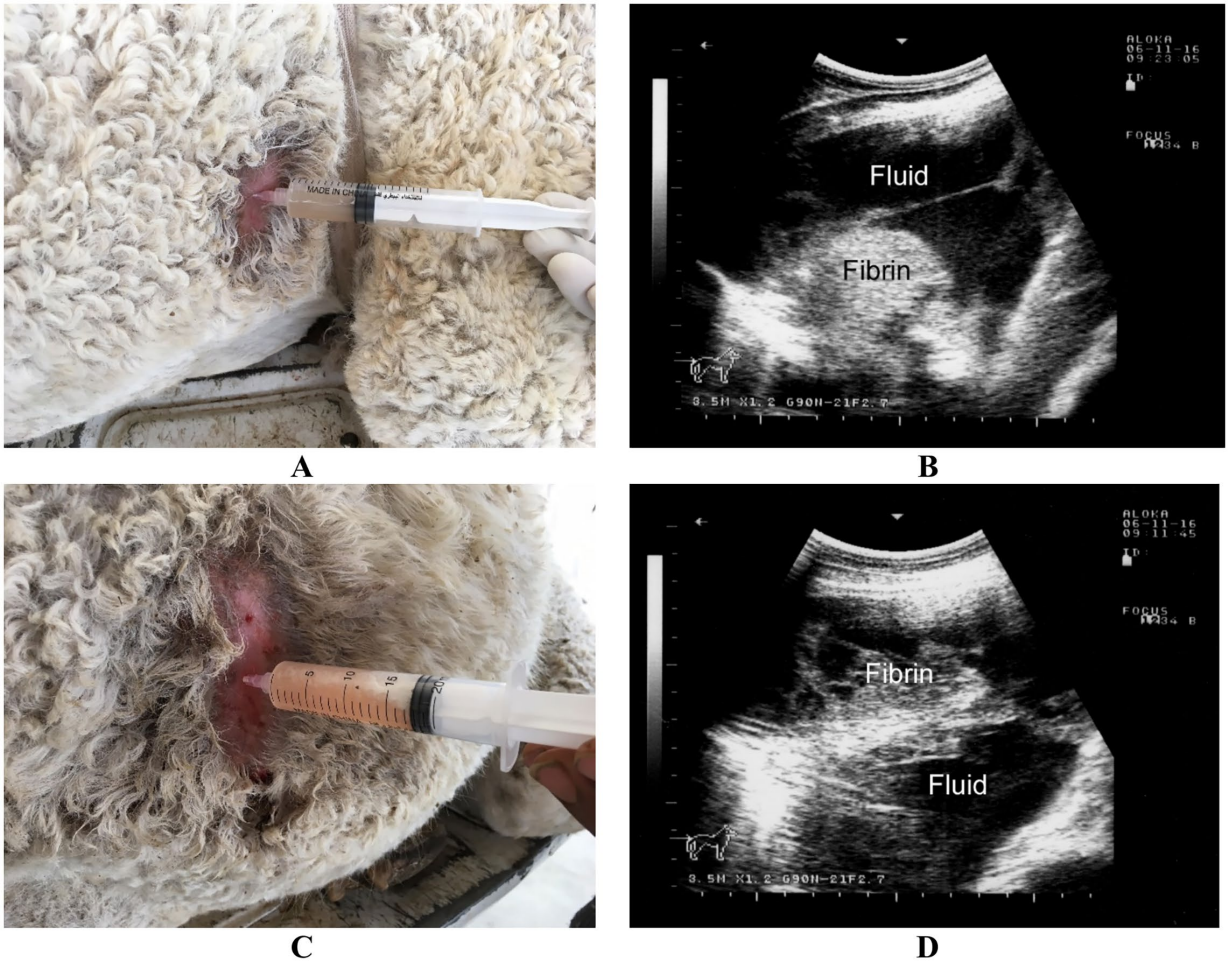
Ultrasound-guided aspiration of pleural exudate in a dromedary camel with pleuropneumonia. **(A)** Shows the aspiration procedure performed on the right thoracic side, while **(C)** depicts the procedure on the left side. Ultrasonographic images of the thoracic cavity are shown in **(B)** and **(D)**, revealing pleural fluid accumulation along with fibrin deposition on both the right and left sides, respectively. Adapted from Tharwat (28), with permission by Qassim University.

**Figure 2:**
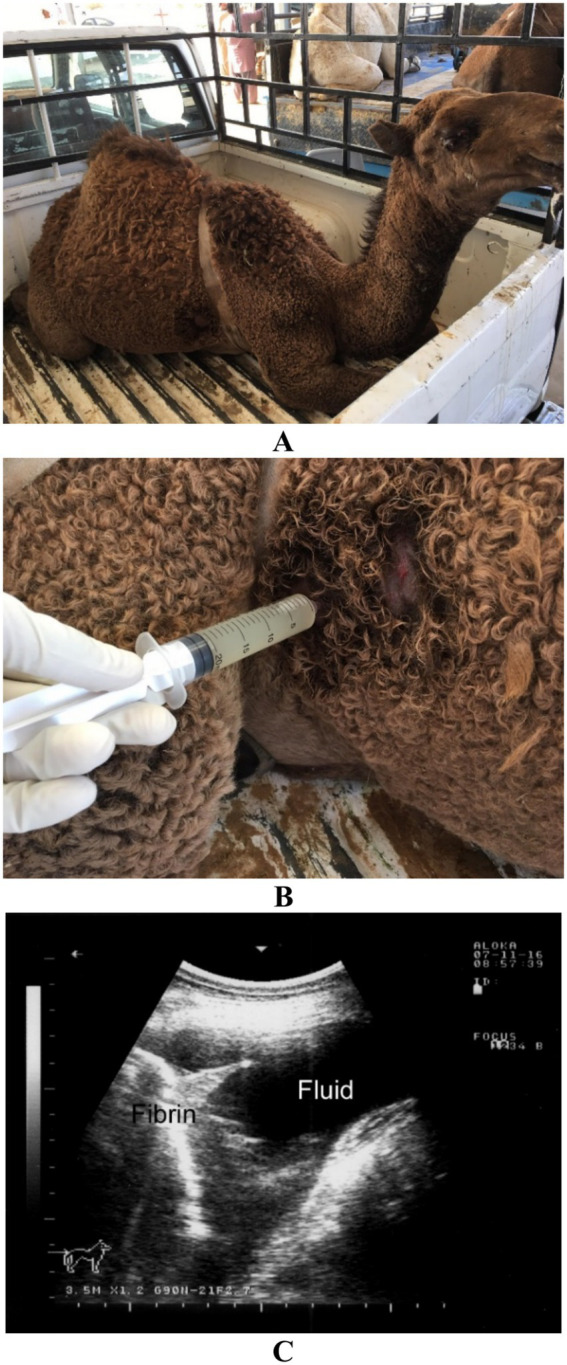
Ultrasound-guided aspiration of pleural exudate in a dromedary camel with pleuropneumonia. The animal presented with fever and depression **(A)**. Ultrasound-guided aspiration yielded turbid pleuritic fluid **(B)**. Image **(C)** shows an ultrasonogram of the right pleural cavity, highlighting pleural fluid accumulation and fibrin deposition. Adapted from Tharwat (28), with permission by Qassim University.

Ultrasound-guided thoracocentesis is an essential procedure in cases where pleural fluid accumulation compromises respiratory function or requires diagnostic analysis. In camels, pleural effusion may arise from infectious, traumatic, or neoplastic causes (37, 38). Ultrasound facilitates safe access to the pleural space by allowing precise localization of fluid collections and real-time needle guidance for further pleural fluid examinations (28) (Figures 1, 2). The probe is typically positioned between the 7th and 9th intercostal spaces, with the transducer oriented longitudinally to visualize the fluid pocket. A 14-gauge sterile needle is introduced cranial to the rib margin to avoid the intercostal vessels and directed into the fluid space under continuous ultrasound observation. The aspirated material is subsequently subjected to cytology, culture, and pleural pressure analysis, aiding in the differentiation of transudates, exudates, or hemorrhagic effusions (34). Despite its benefits, thoracocentesis carries risks, including pneumothorax, pulmonary laceration, and hemorrhage, particularly when anatomical landmarks are obscured or the animal moves unexpectedly (33). Ultrasound guidance substantially reduces these risks by providing dynamic imaging during needle advancement; however, it does not completely eliminate the possibility of complications (28, 32).

### 3.2 Abdominocentesis

**Figure 3:**
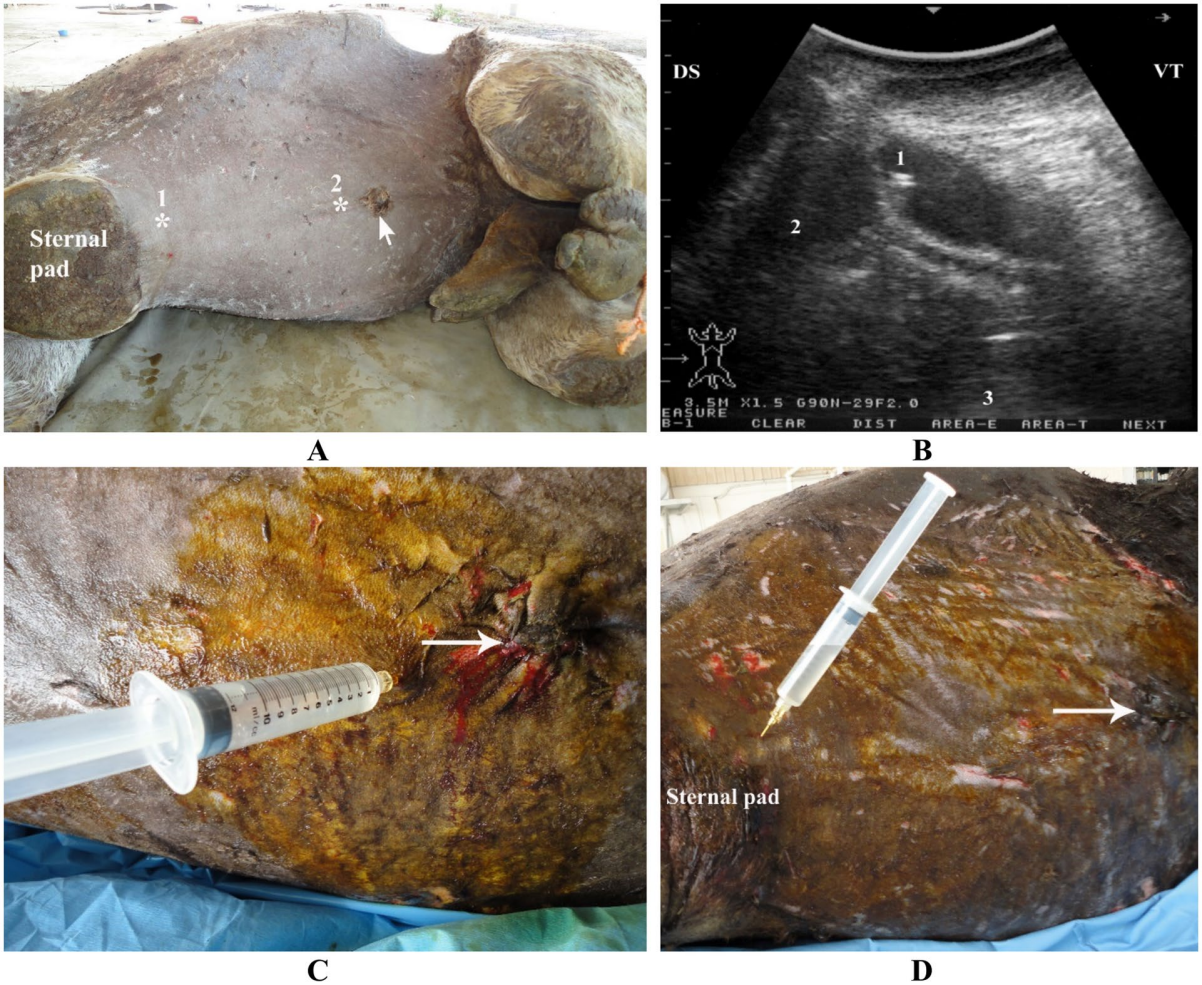
Ultrasound-guided abdominocentesis in a dromedary camel. Image **(A)** shows the aspiration sites marked with stars; the hair was clipped, the skin shaved, and the animal positioned laterally. Two sites were selected for abdominocentesis: 10 cm caudal to the sternal pad (1) and 10 cm cranial to the umbilicus (2). Image **(B)** depicts the ultrasound-guided procedure at the site 10 cm caudal to the sternal pad, showing a contracted reticulum. Labels indicate the needle (1), peritoneal fluid (2), and reticular wall (3). Images **(C)** and **(D)** show aspiration of clear peritoneal fluid from the anterior site (10 cm caudal to the sternal pad) and posterior site (10 cm cranial to the umbilicus), respectively. Arrows indicate the umbilicus. Adapted from Tharwat et al. (44), with permission from Elsevier.

In human medicine, ultrasound plays a pivotal role by identifying optimal sites for fluid collection and minimizing complications associated with blind aspiration techniques (39, 40). In animals, abdominal paracentesis is frequently employed in the evaluation of peritoneal effusion, suspected peritonitis, and abdominal trauma (41–43). In camels, the ultrasonographic examination begins with a systematic survey of the ventral abdomen to detect anechoic or hypoechoic fluid accumulations. Once located, the site of abdominocentesis is aseptically prepared, and the needle is introduced under direct visualization to avoid penetrating adjacent viscera such as the intestines (Figure 3) (44). While generally safe, potential complications include inadvertent bowel puncture, iatrogenic peritonitis, and localized hemorrhage (33). The risk is significantly lowered when fluid pockets are clearly delineated and when the operator maintains a steady hand during the needle’s advancement (28).

### 3.3 Portocentesis

Portocentesis, or the sampling of blood directly from the portal vein, is a specialized diagnostic procedure used to evaluate hepatic hemodynamics and investigate suspected portal hypertension or liver dysfunction (45–47). Collecting blood from the portal vein in large ruminants offers important research and diagnostic benefits (48, 49). It provides direct access to blood from the gastrointestinal tract, enabling more accurate assessment of nutrient absorption and hepatic metabolism before substances enter systemic circulation (50, 51). This approach is particularly valuable for evaluating feed component bioavailability and understanding how the liver processes nutrients, toxins, or medications (52). Additionally, portal vein sampling can aid in the early detection of digestive or liver disorders by revealing abnormal metabolite profiles that might not be apparent in peripheral blood (53).

**Figure 4:**
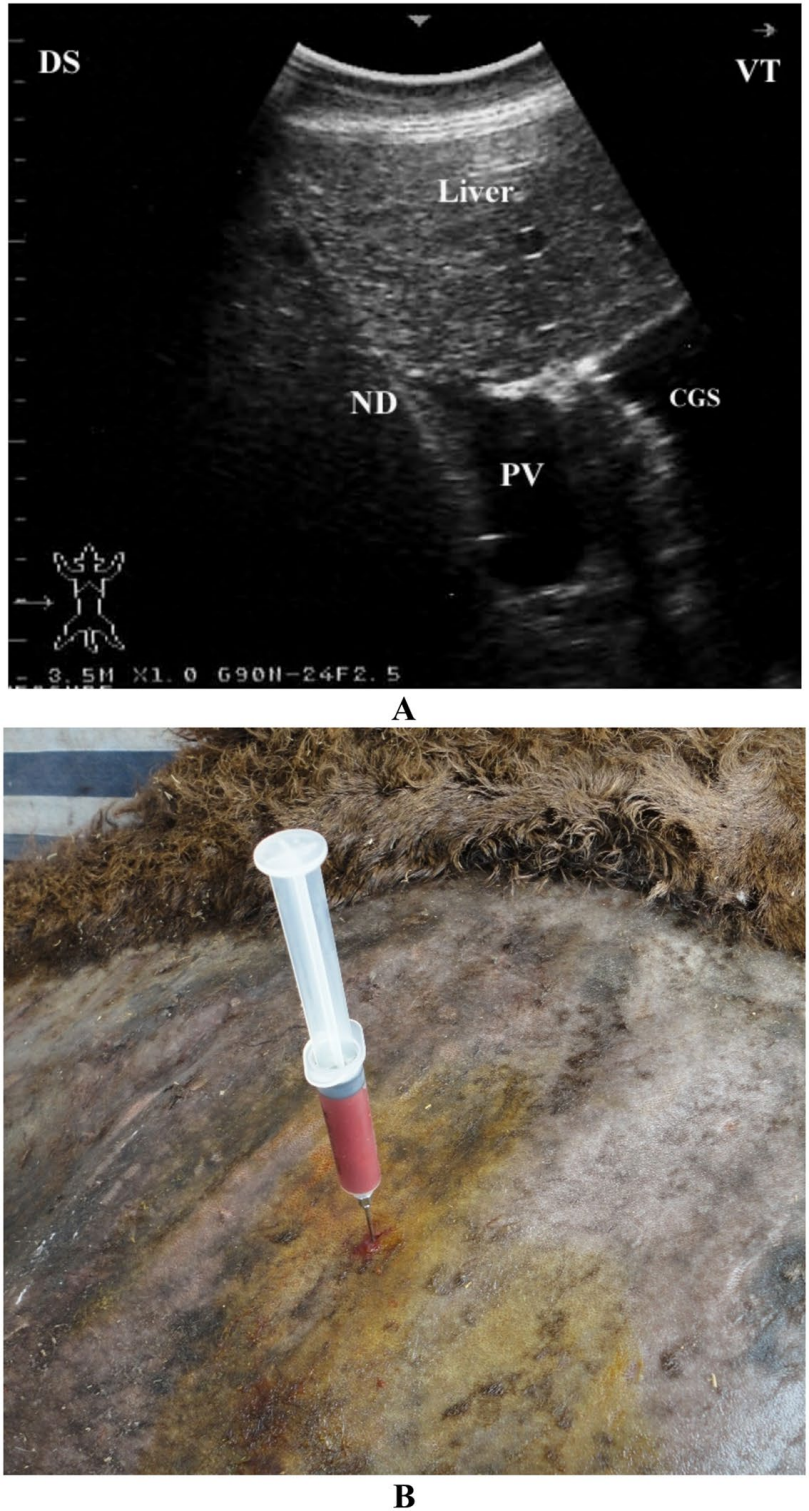
Ultrasound-guided portocentesis in a dromedary camel. The needle (ND) appears on the ultrasound screen as a sharp, bright line. Image **(A)** was captured in the right 10th intercostal space using a 3.5 MHz convex transducer. Image **(B)** shows 20 mL of portal blood collected under ultrasound guidance. PV, portal vein; CGS, caudal glandular sacs; DS, dorsal; VT, ventral. Adapted from Tharwat et al. (54), with permission by Journal of Camel Practice and Research.

In dromedary camels, where hepatic pathology can be clinically silent, this technique offers a unique opportunity for direct vascular assessment (54). The portal vein appears triangular in healthy camels, and is visualized using a transabdominal approach with the camel in sternal or lateral recumbency (21, 55). Once identified, a fine-gauge needle is guided into the vessel under real-time ultrasound control, ensuring accurate placement while minimizing the risk of inadvertent injury to adjacent organs or vasculature (Figure 4) (54). Although hemorrhage and vascular injury remain potential complications, these are rare with proper technique and the use of high-resolution imaging equipment (52). Portocentesis, when performed correctly, offers valuable information for hepatic disease management that cannot be obtained through peripheral blood analysis alone (28).

### 3.4 Ultrasound-guided biopsies

**Figure 5:**
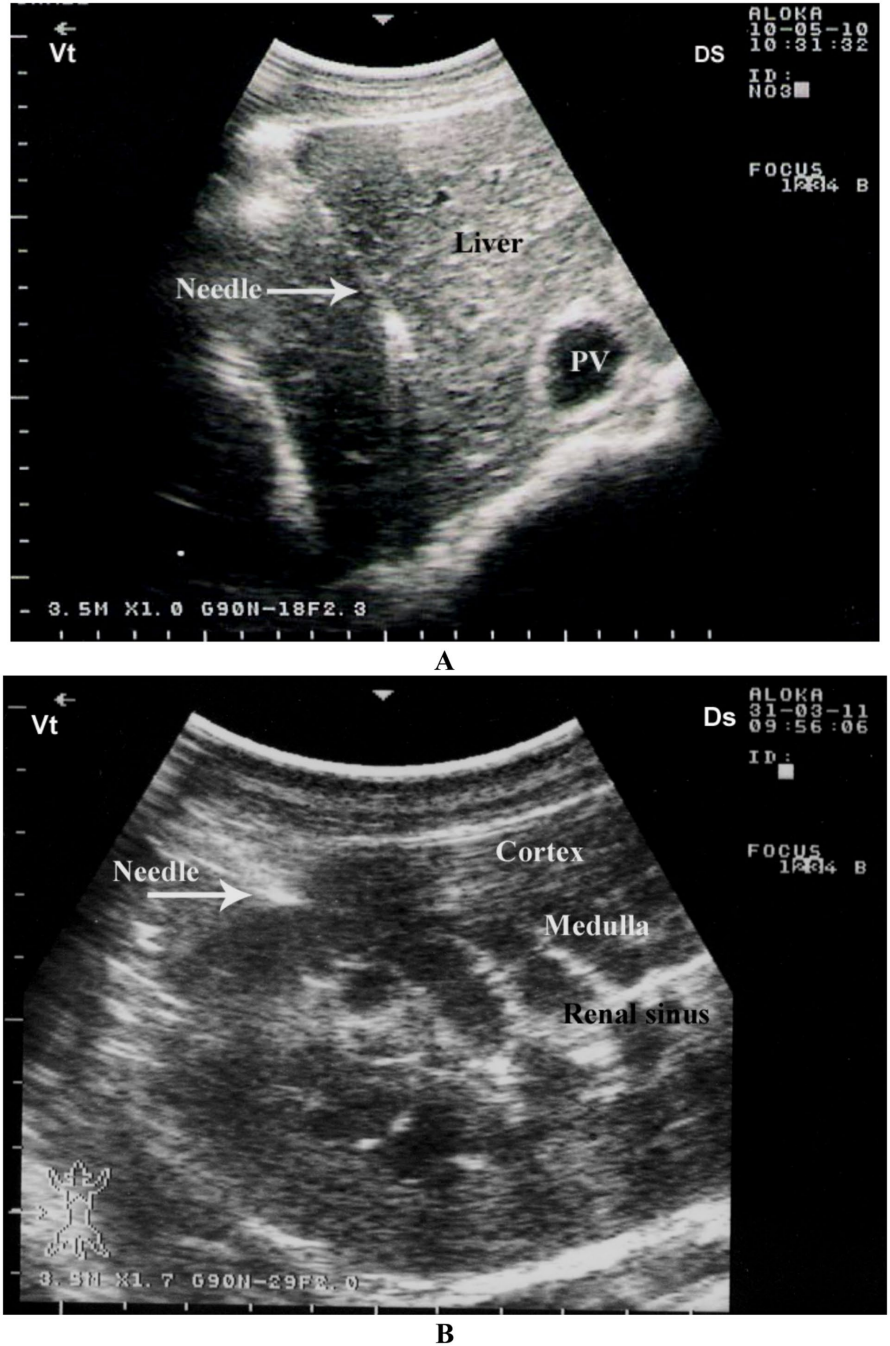
Fine-needle biopsy of hepatic and renal tissue in a dromedary camel. The needle is clearly visible as a sharp, bright line within the hepatic tissue **(A)** and renal tissue **(B)**. PV, portal vein; Ds, dorsal; Vt, ventral. Modified from Tharwat et al. (30), with permission from Elsevier.

Ultrasound-guided biopsy is a minimally invasive yet highly informative procedure used to characterize hepatic, renal, and intra-abdominal lesions in camels (30). It is particularly useful when there is a need to differentiate between neoplastic, inflammatory, and metabolic causes of organ dysfunction (56, 57). For hepatic and renal biopsies, ultrasound enables selection of the most accessible and representative area of the organ, facilitating either core biopsy or fine-needle aspiration depending on lesion size, depth, and suspected pathology. The procedure of ultrasound-guided liver and kidney biopsy is safe, fast, cost-effective, and practical in camels as long as it is performed properly (30). The needle was consistently visualized on the ultrasound monitor within the hepatic parenchyma and renal cortex as a distinct, bright linear structure (Figure 5).

**Figure 6:**
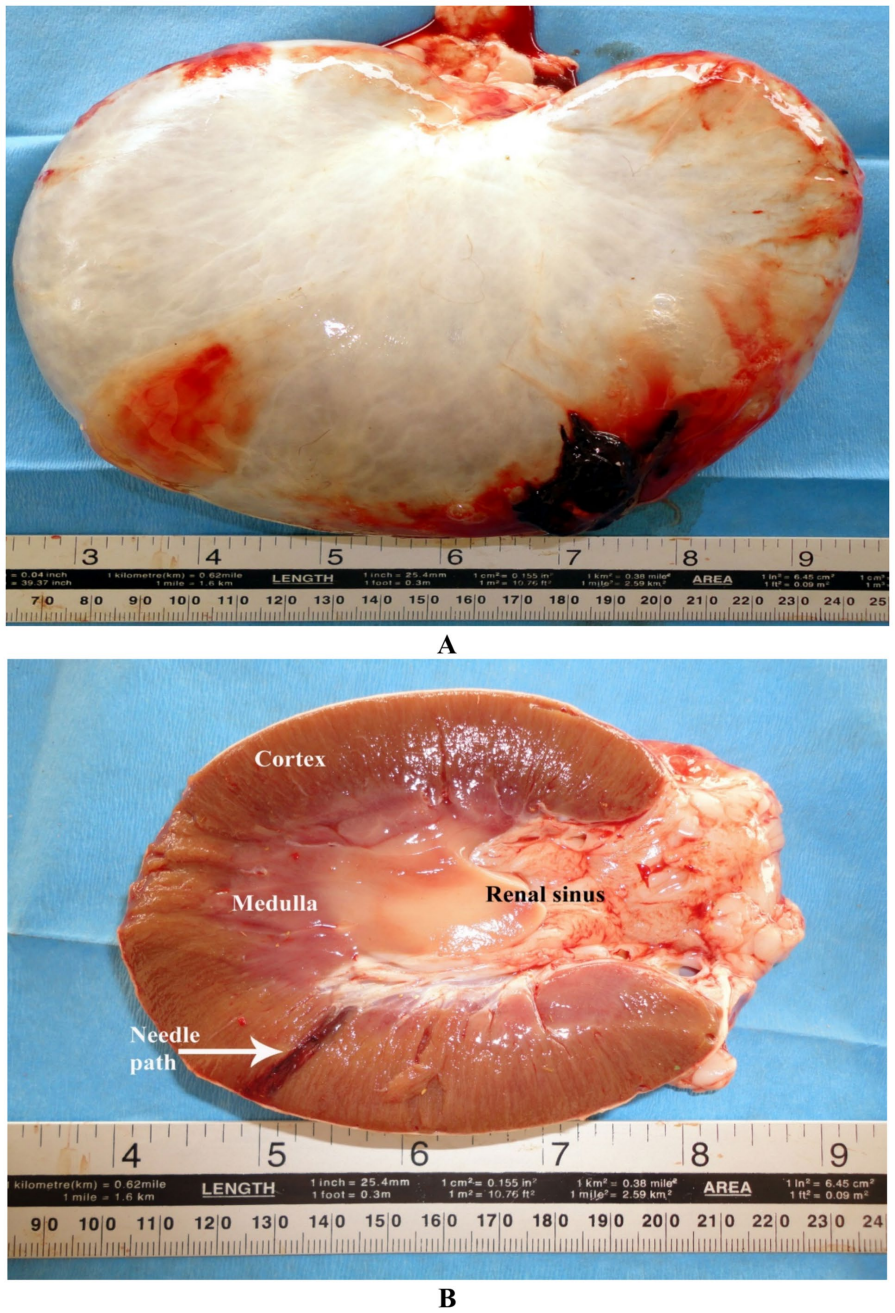
Post-renal biopsy complication in a dromedary camel. A subcapsular hematoma was observed immediately after the procedure **(A)**. At postmortem examination, the biopsy needle path was clearly identified within the renal cortex **(B)**. Modified from Tharwat et al. (30), with permission from Elsevier.

Abdominal masses—whether tumors, granulomas, or abscesses—can also be safely sampled using flank or ventral abdominal approaches. Real-time imaging ensures accurate needle trajectory while avoiding critical structures (56, 57). Renal complications, though infrequent, may include hemorrhage, infection, or the formation of subcapsular hematoma, especially if biopsy protocols are not meticulously followed (Figure 6) (30). Ultrasound-guided biopsies thus represent a critical component of modern camelid diagnostics, providing actionable data while preserving animal safety and welfare (33).

## 4 Ultrasound-guided therapeutic procedures

Ultrasound-guided interventions have become essential tools in modern human and veterinary medicine, offering minimally invasive, targeted therapeutic options for a range of internal conditions (58–60). In dromedary camels (*Camelus dromedarius*), these techniques are particularly valuable given the species’ anatomical distinctiveness, resilience to overt clinical signs, and the challenges associated with conventional surgical approaches (61). The use of ultrasound not only enables precise localization of pathological lesions but also enhances safety and efficacy during therapeutic procedures (28). This section highlights two of the most clinically relevant ultrasound-guided interventions in camelid practice: relief of pleural effusion and abscess drainage.

### 4.1 Relief of pleural effusion

Pleural effusion, characterized by the accumulation of fluid in the pleural space, poses a significant threat to respiratory function in camels (62). Clinically, affected animals may present with tachypnea, reduced thoracic excursions, and audible fluid sounds on auscultation (63). Ultrasound is pivotal in both diagnosing and guiding the treatment of pleural effusion, allowing for real-time visualization of fluid pockets and adjacent thoracic structures (33).

**Figure 7:**
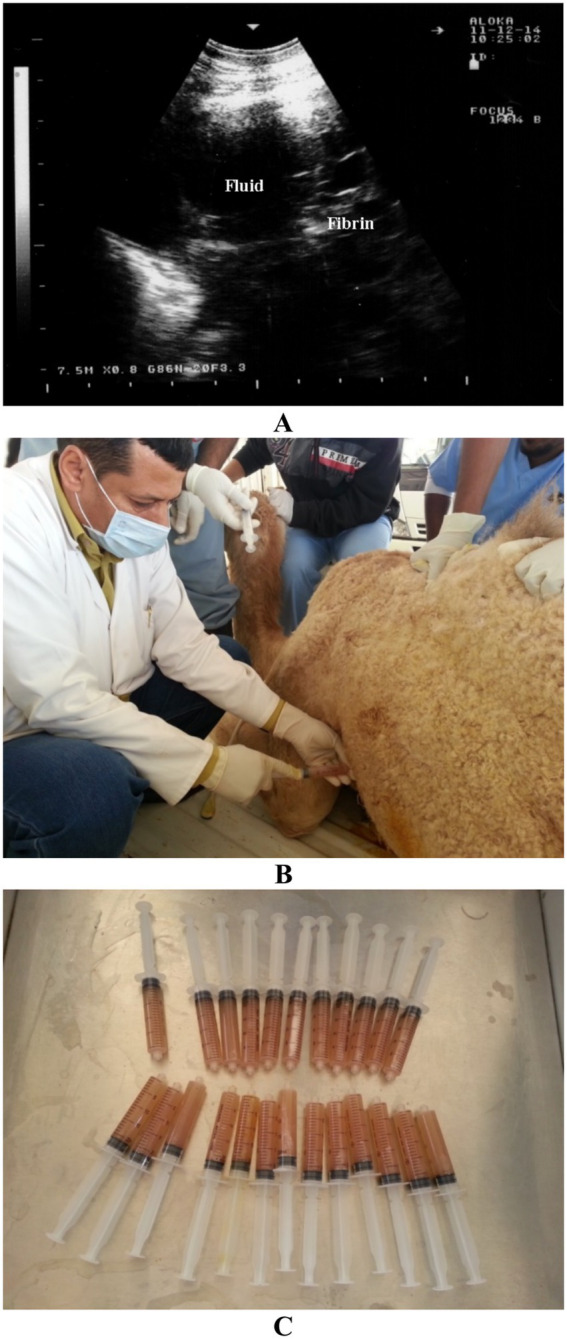
Thoracic ultrasonography in a female camel with pleuropneumonia showing anechoic fluid with a fibrin net within the pleura **(A)**. Thoracocentesis of the left pleural sac is shown in **(B)**, with approximately 500 mL of aspirated pleural exudate in **(C)**. Adapted from Tharwat (38), licensed under CC BY 4.0.

The primary therapeutic goal is the alleviation of respiratory distress through controlled thoracocentesis or catheter-based drainage. Once the effusion is confirmed sonographically, the operator identifies the optimal site for needle or catheter insertion, typically at the ventral thoracic margin where fluid accumulates (30) (Figure 7). Under aseptic conditions, and using in-plane or out-of-plane guidance techniques, a sterile catheter is introduced into the pleural cavity. In many cases, continuous or intermittent drainage may be required, necessitating secure placement of a pigtail or thoracostomy catheter (28). The volume and character of the drained fluid should be monitored carefully, as rapid removal may precipitate re-expansion pulmonary edema or hemodynamic instability (34).

### 4.2 Drainage of internal abscessations (renal, hepatic, retroperitoneal)

**Figure 8:**
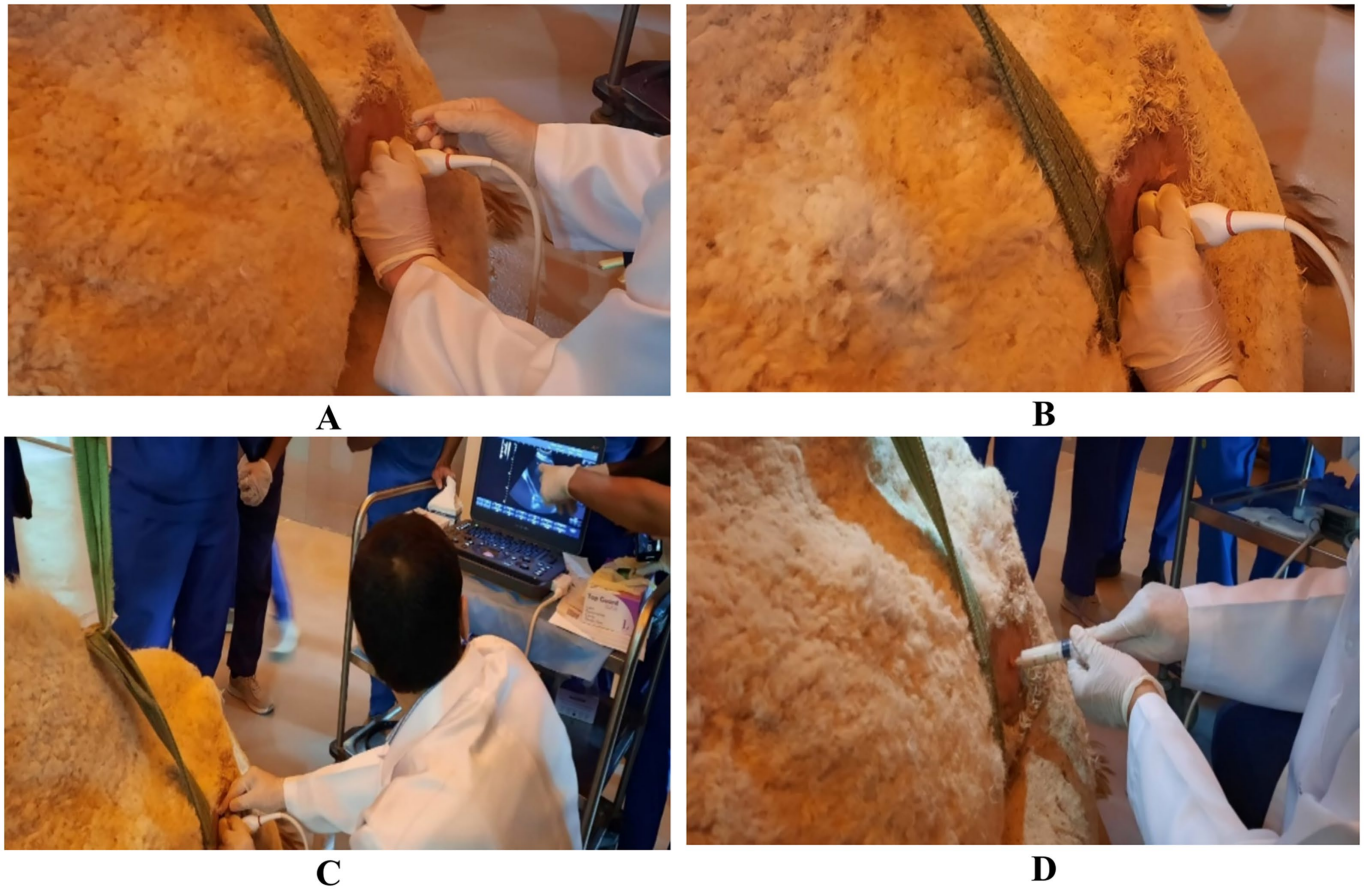
Ultrasound-guided aspiration of abscess contents in the left kidney of a dromedary camel. Image **(A)** shows the site, with the needle positioned perpendicular to the transducer **(B)** before being inserted into the center of the lesion **(C)**. Finally, the pus sample was collected **(D)**. Modified from Tharwat et al. (29), with permission by Open Veterinary Journal.

**Figure 9:**
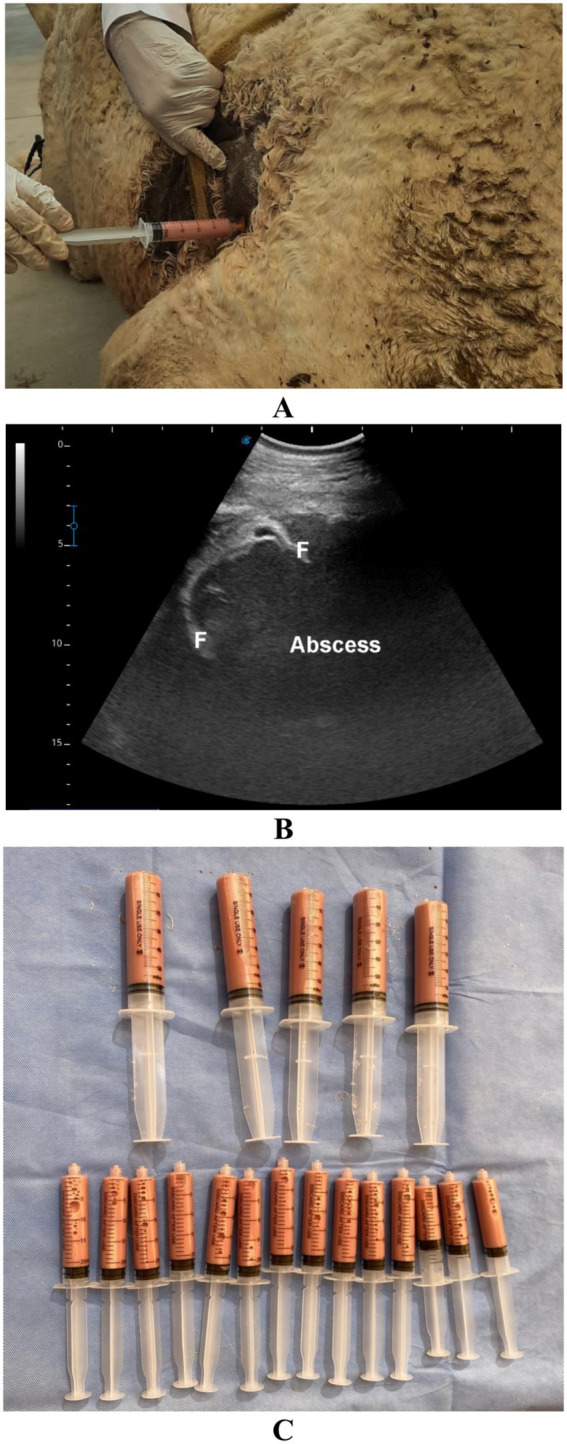
Retroperitoneal abscess in a dromedary camel presenting with a 4-month history of gradual body weight loss. Image **(A)** shows ultrasound-guided aspiration of the abscess, while image **(B)** reveals hyperechogenic contents of the lesion with fibrin threads (F). Aspiration of the lesion yielded 600 mL of pus **(C)**. Adapted from Tharwat (28), with permission by Qassim University.

Ultrasound also plays a critical role in the management of internal abscesses, particularly those involving parenchymal organs such as the liver and kidneys, or deep-seated retroperitoneal tissues (19–21). These abscesses are frequently sequelae to hematogenous infections or penetrating trauma and may remain clinically occult until reaching considerable size (32). Once an abscess is identified on ultrasound—typically as a hypoechoic to anechoic cavity with internal septations or debris—therapeutic drainage becomes a viable option. Real-time sonographic guidance ensures accurate needle placement into the abscess core, minimizing injury to surrounding vasculature or parenchyma (Figure 8) (29). Aspiration of purulent material is followed, in many cases, by lavage using sterile isotonic saline to evacuate residual debris (28) (Figure 9). In some scenarios, placement of a drainage catheter may be warranted for repeated flushing or prolonged drainage (33, 34). While ultrasound-guided drainage is considerably safer than blind aspiration or surgical excision, it is not without risk. Complications may include incomplete evacuation of the abscess cavity, introduction or exacerbation of systemic infection, and accidental damage to adjacent organs or vessels (28). Thus, close post-procedural monitoring and adjunctive antimicrobial therapy are essential to optimize outcomes.

## 5 Differentiating the nature of ascites based on ultrasound guidance results

Accurate characterization of ascitic fluid plays a vital role in the diagnosis and management of underlying pathological conditions in dromedary camels (64). Ultrasound-guided evaluation has emerged as a pivotal diagnostic modality in camelid medicine, offering a non-invasive, real-time assessment of abdominal effusion and facilitating targeted fluid sampling (32). While the presence of ascites in camels may be indicative of a wide range of etiologies, ultrasound guidance results enhance the clinician’s ability to distinguish between transudative, exudative, and bloody effusions prior to laboratory confirmation (28).

### 5.1 Ultrasound features of ascitic fluid

On ultrasonographic examination, ascitic fluid typically appears as an anechoic or hypoechoic accumulation within the peritoneal cavity (65). The echogenicity of the fluid can provide preliminary clues to its composition (66). Clear, transudative ascites tends to be uniformly anechoic; in contrast, exudative or inflammatory effusions may present with internal echoes, septations, or fibrin strands (67). Bloody ascites—hemoperitoneum—frequently demonstrates heterogeneous echogenicity with swirling echogenic foci, indicating the presence of blood clots or active bleeding (68). In dromedary camels, normal peritoneal fluid is either absent or present in trace amounts and appears as anechoic on ultrasound. Ascitic fluid becomes sonographically visible when its volume exceeds 100–200 mL, typically accumulating between intestinal loops, around the liver, or in the subxiphoid and paralumbar regions (44).

### 5.2 Ultrasound-guided aspiration for differentiating serous and hemorrhagic ascites

**Figure 10:**
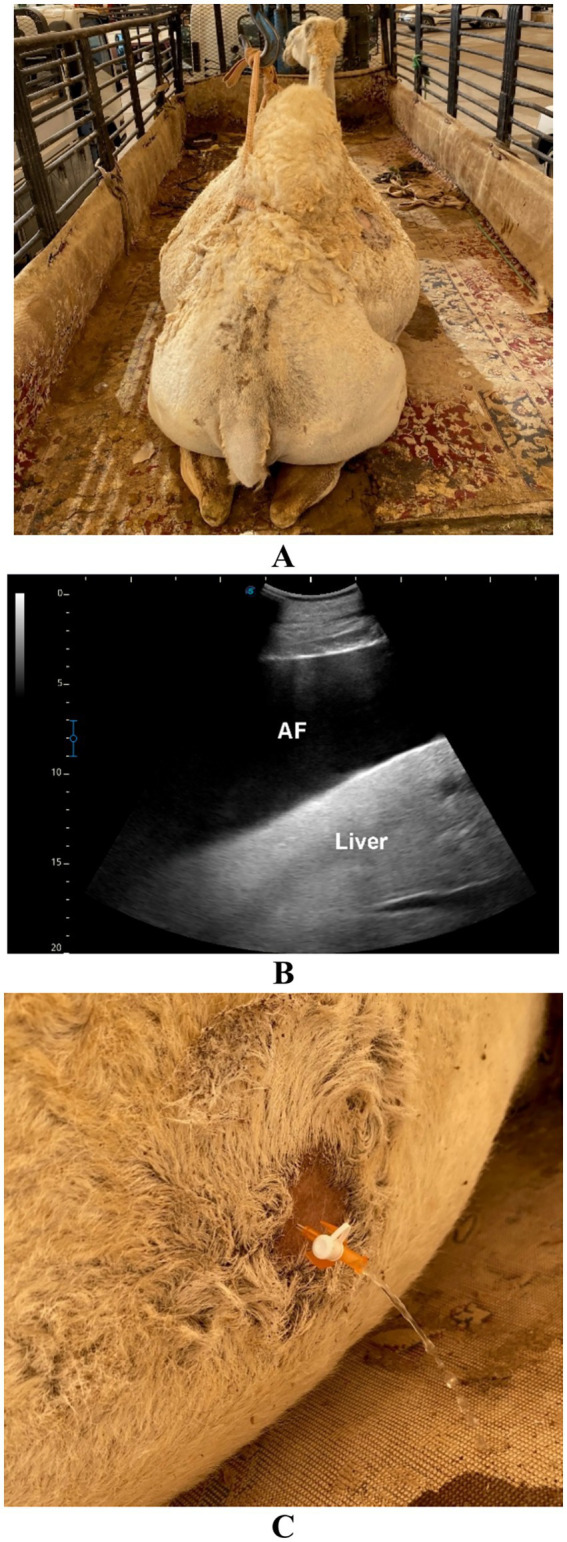
Ascites in a female dromedary camel. **(A)** The camel presented with a two-month history of progressive bilateral abdominal distension. **(B)** Ultrasound image showing anechoic ascitic fluid (AF) accumulation within the peritoneal cavity. **(C)** Ultrasound-guided aspiration of clear to straw-colored fluid. Adapted from Tharwat and Al-Sobayil (34), with permission by Qassim University.

**Figure 11:**
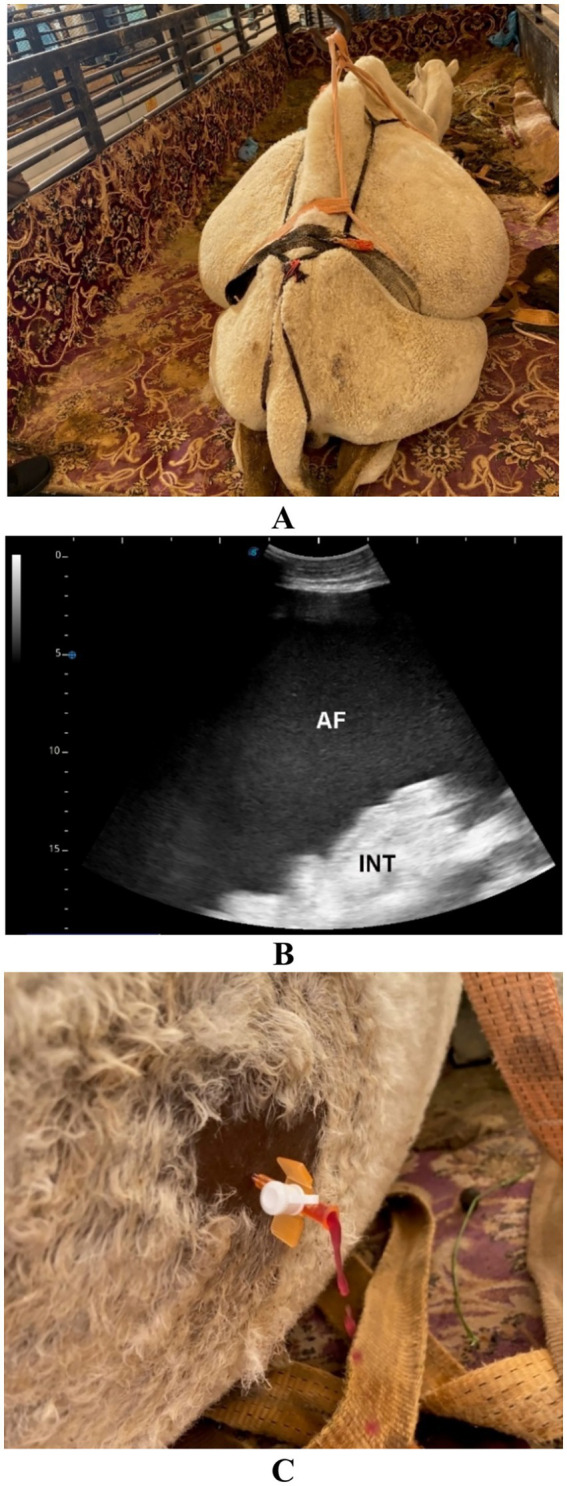
Ascites in a female dromedary camel. **(A)** The camel was admitted with a four-month history of progressive bilateral abdominal distension. **(B)** Ultrasound image showing echogenic ascitic fluid (AF) accumulation within the peritoneal cavity where intestines (INT) are floating. **(C)** Ultrasound-guided aspiration of blood-tinged fluid. Adapted from Tharwat and Al-Sobayil (33), with permission by Qassim University.

Ultrasound-guided aspiration significantly enhances the accuracy and safety of ascitic fluid sampling in dromedary camels, particularly when distinguishing between serous and hemorrhagic effusions (34). While B-mode ultrasound alone can suggest the fluid’s nature based on echogenicity, aspiration under real-time guidance allows definitive assessment through direct visual inspection and laboratory evaluation (28). Serous or transudative ascites is typically clear to straw-colored and cytologically low in protein and cellularity, often reflecting hypoalbuminemia (19) (Figure 10). In contrast, hemorrhagic ascites presents as blood-tinged to frankly bloody fluid and may yield clotted or unclotted samples depending on the chronicity and etiology of the hemorrhage (Figure 11). Sonographically, hemorrhagic fluid often correlates with echogenic swirling or sedimentation, yet overlap can occur with protein-rich exudates or septic peritonitis (69). Therefore, ultrasound-guided aspiration is not only crucial for obtaining diagnostic-quality fluid from localized pockets but also minimizes the risk of iatrogenic injury, particularly in camels where abdominal viscera are deeply positioned and patient restraint is complex (32). In cases of suspected hemoperitoneum—such as following trauma or uterine rupture—ultrasound guidance allows targeted sampling away from active bleeding sites, enhancing both diagnostic safety and yield (28).

### 5.3 Clinical relevance

Understanding the ultrasonographic patterns of bloody versus non-bloody ascites is critical in guiding further diagnostic workup and therapeutic intervention (70). For example, detection of hemoperitoneum may warrant immediate surgical exploration such as ruptured urinary bladder in male dromedaries (71) while inflammatory ascites could indicate peritonitis necessitating antimicrobial therapy (72). Thus, ultrasound-guided assessment not only refines diagnostic accuracy but also directly influences clinical decision-making in camelid practice.

## 6 Ultrasound-guided differentiation of uroperitoneum

In dromedary camels, the accurate diagnosis of uroperitoneum—a condition defined by the accumulation of urine within the peritoneal cavity—remains clinically challenging due to the vague and non-specific clinical presentation (73). This condition may arise from either urinary bladder rupture or leakage from an otherwise intact urinary tract due to obstruction, increased intravesical pressure, or traumatic injury (34). Timely differentiation between these etiologies is critical, as it guides therapeutic decision-making and surgical planning. Among the available diagnostic tools, ultrasound-guided abdominal paracentesis offers a safe, minimally invasive method for obtaining peritoneal fluid samples while concurrently providing real-time visualization of intra-abdominal structures (28).

### 6.1 Ultrasonographic visualization of peritoneal fluid

**Figure 12:**
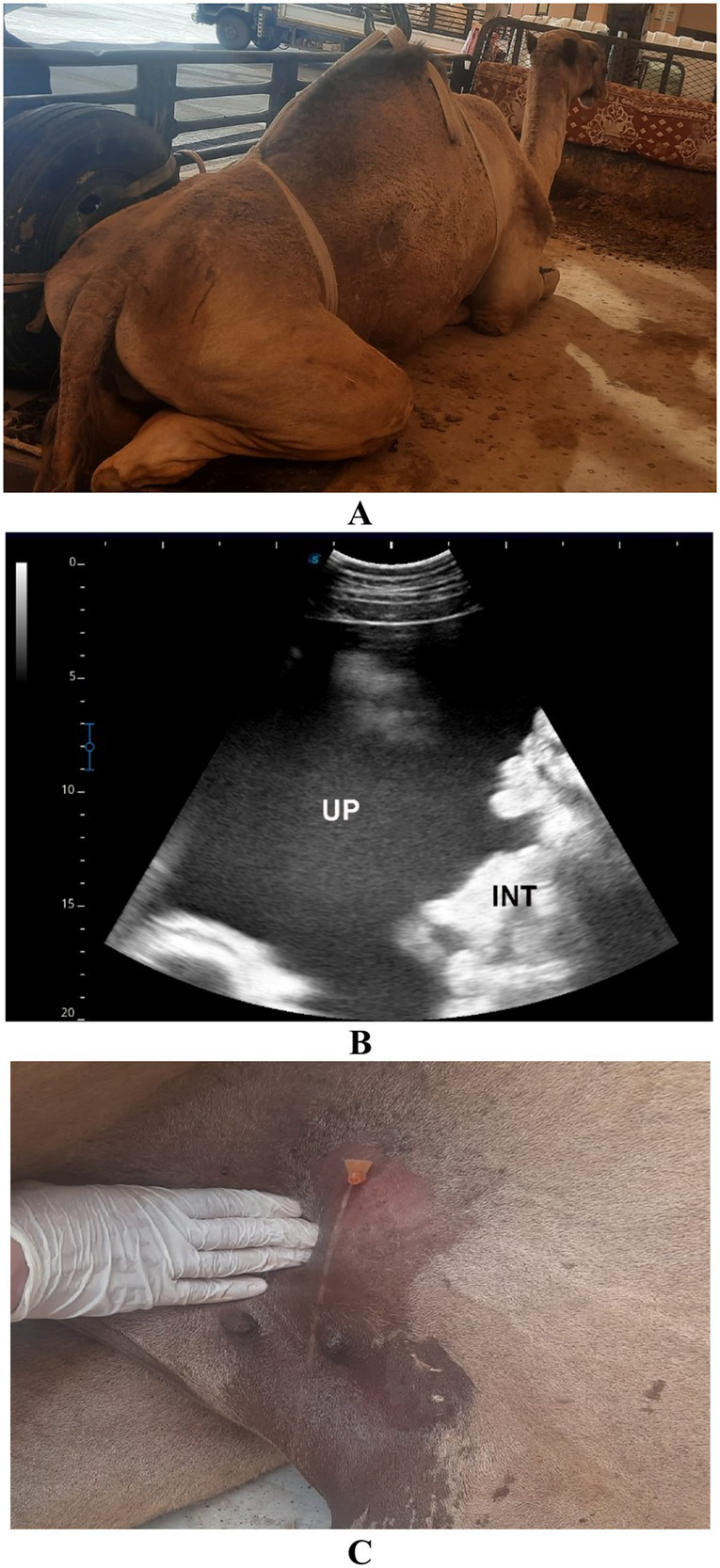
Urine retention in a male dromedary camel presented with a 5-day history of abdominal pain, anorexia, and anuria. Image **(A)** shows the clinical appearance of the animal. Ultrasonographic examination **(B)** reveals echogenic uroperitoneum (UP) with floating intestinal loops (INT), indicative of urine accumulation in the abdominal cavity. Image **(C)** displays the turbid appearance of the aspirated uroperitoneal fluid. Adapted from Tharwat and Al-Sobayil (33), with permission by Qassim University.

**Figure 13:**
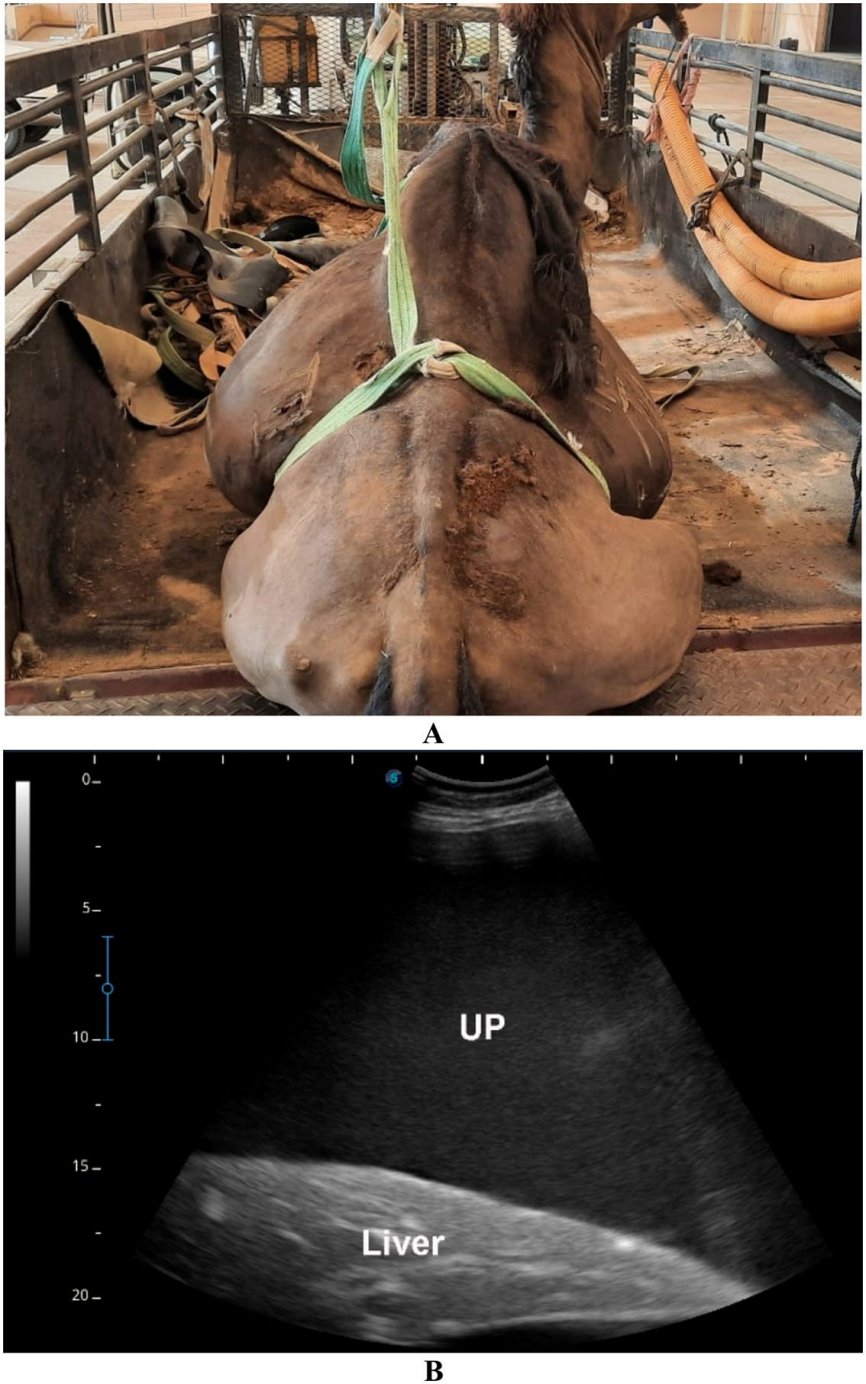
Urine retention in a male dromedary camel presented with a 10-day history of gradual abdominal distension, abdominal pain, anorexia, and anuria. Image **(A)** shows the clinical presentation of the animal. Ultrasonographic image **(B)** reveals echogenic uroperitoneum (UP) with the liver seen floating within the fluid. Adapted from Tharwat and Al-Sobayil (33), with permission by Qassim University.

Anechoic or mildly echogenic free fluid is typically visualized between the intestinal loops, in the caudoventral abdomen, or around the urinary bladder (28). The echogenicity of the fluid may vary depending on the chronicity of the condition or the presence of secondary inflammation. The most accessible sites for fluid aspiration are the right or left caudoventral abdominal quadrant, lateral to the *linea alba* and cranial to the udder or prepuce (28). After identifying a fluid pocket, a spinal needle or echogenic catheter is introduced under direct sonographic guidance to aspirate the peritoneal fluid (33). This technique minimizes complications such as inadvertent puncture of abdominal organs or blood vessels. Clear to straw-colored fluid is usually aspirated in camels with urine retention but with intact urinary bladder (Figure 12). In contrast, in cases of urinary bladder rupture, the aspirated uroperitoneum appears hemorrhagic (Figure 13) (28).

### 6.2 Clinical implications and decision-making

Ultrasound-guided aspiration serves a dual diagnostic function: it confirms the presence of peritoneal effusion and provides fluid for laboratory analysis, enabling precise etiological categorization (28). Prompt identification of bladder rupture mandates surgical intervention, whereas uroperitoneum due to functional obstruction may respond to urinary catheterization, anti-inflammatory therapy, and correction of underlying causes (37). As such, this imaging-guided technique is invaluable in reducing diagnostic uncertainty and optimizing patient outcomes (34).

## 7 Ultrasound-guided aspiration of peritoneal effusions in camels with peritonitis

Peritonitis in dromedary camels, whether acute or chronic, is a significant cause of morbidity and can manifest clinically as abdominal distension, pain, and systemic signs such as fever or lethargy (63). While ultrasonography is instrumental in identifying intra-abdominal effusions and guiding clinical suspicion, ultrasound-guided aspiration remains the definitive step for diagnostic confirmation and therapeutic planning (32). This minimally invasive procedure allows for precise sampling of peritoneal fluid under real-time imaging, significantly improving diagnostic yield and safety in camels presenting with non-specific abdominal signs (72).

### 7.1 Ultrasonographic features in peritonitis and diagnostic utility of aspirated fluid

**Figure 14:**
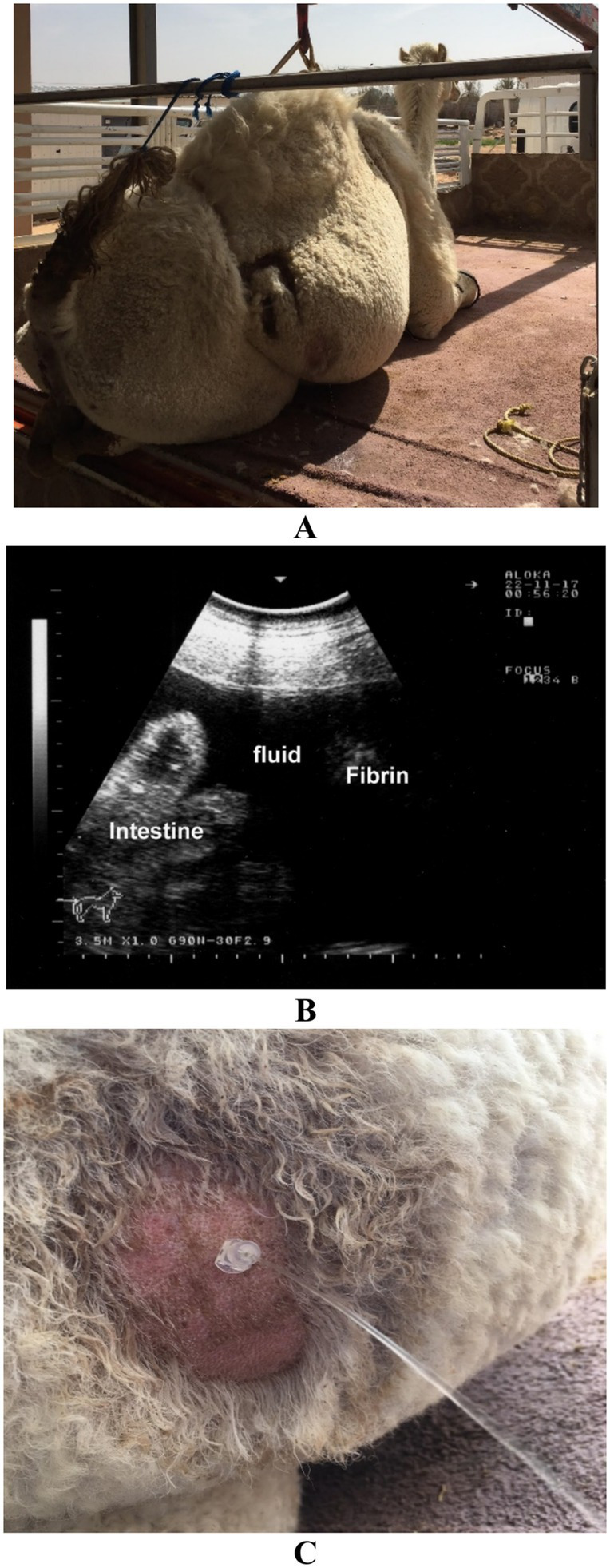
Peritonitis in a female dromedary camel. **(A)** The camel presented with clinical signs of inappetence and bilateral abdominal distension. **(B)** Ultrasonographic examination reveals anechoic peritoneal effusion. **(C)** Ultrasound-guided abdominocentesis demonstrates aspiration of clear peritoneal fluid. Adapted from Tharwat and Al-Sobayil (33), with permission by Qassim University.

**Figure 15:**
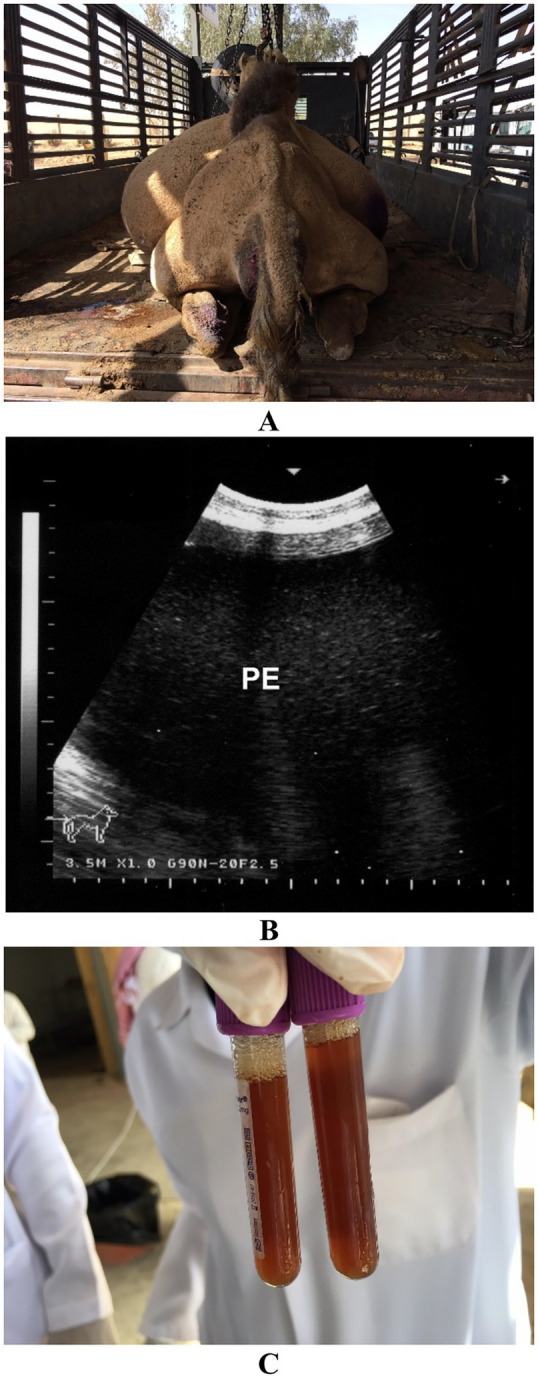
Peritonitis in a female dromedary camel. **(A)** The camel was presented with marked, progressive abdominal distension. **(B)** Ultrasonographic image reveals echogenic peritoneal effusion (PE) suggestive of chronic inflammation. **(C)** Ultrasound-guided abdominocentesis shows bloody peritoneal fluid aspirated from the abdominal cavity. Adapted from Tharwat and Al-Sobayil (33), with permission by Qassim University.

In camels with peritonitis, ultrasonography often reveals echogenic fluid with floating fibrin strands, septations, and visceral adhesions. Chronic peritonitis may show less dramatic findings, with fluid appearing clear, serosanguinous, or occasionally bloody, depending on the degree of inflammation and vascular leakage (72). The nature of aspirated fluid provides valuable clues about the underlying etiology and chronicity of peritonitis. Acute septic peritonitis typically yields clear or turbid exudate with high protein content and neutrophilic predominance on cytology (Figure 14) (19). In contrast, chronic peritonitis may yield clear or serosanguinous fluid with lower cellularity and occasional macrophage-rich profiles (Figure 15) (28).

## 8 Limitations of ultrasound-guided interventions in camels

Ultrasound-guided procedures in adult dromedary camels are constrained by anatomical, environmental, procedural, and evidentiary factors. The large body size and depth of internal organs beneath thick musculature and adipose tissue frequently limit image resolution and penetration, even when low-frequency transducers (2–5 MHz) are used, particularly in obese or dehydrated animals (19–21, 28, 33, 34). Field application is further challenged in remote pastoral settings by limited access to power sources, suboptimal environmental control, and difficulty maintaining sterility during invasive procedures, increasing the risk of complications (32, 74). Sedation, often required for safe intervention, may be contraindicated in systemically compromised camels, as commonly used agents such as xylazine can exacerbate cardiopulmonary or hemodynamic instability (28, 75). Procedural success is highly dependent on operator expertise, and inadequate training may lead to diagnostic errors or iatrogenic injury, emphasizing the need for structured, camel-specific training (76–78). Finally, despite encouraging case reports and retrospective studies, the lack of prospective, comparative trials limits definitive conclusions regarding efficacy, safety, and long-term outcomes.

## 9 Future directions

Future advances in ultrasound-guided diagnostics and interventions in dromedary camels should focus on addressing current limitations through species-specific adaptation, technological innovation, and improved accessibility (33). Standardization of techniques tailored to camel anatomy is a critical priority, as many existing approaches are extrapolated from other large-animal models and may not adequately reflect camel-specific anatomical features; the development of unified protocols, sonographic landmarks, and structured training within veterinary education is therefore essential, particularly in remote and arid regions (28, 79, 80). Emerging technologies, including AI-assisted ultrasound systems with machine learning–enabled smart probes, offer promising opportunities for automated lesion detection, measurement, and improved image acquisition, as demonstrated in human and small animal medicine, but remain largely unexplored in camelid and large-animal practice (81–88). In parallel, tele-guided ultrasound may enhance diagnostic capacity in geographically dispersed camel populations by enabling real-time remote supervision and interpretation, an approach that has proven effective in human healthcare and holds particular promise for improving early disease detection in underserved regions (89–92).

## 10 Conclusion

Ultrasound-guided diagnostic and therapeutic interventions mark a significant advancement in the veterinary care of dromedary camels, providing minimally invasive, safe, and precise alternatives to traditional methods. Due to the unique anatomy and physiology of camels, ultrasonography is especially effective for clinical challenges in this species. It enhances diagnostic accuracy in conditions such as pleural and peritoneal effusions and supports guided procedures like aspirations, biopsies, and abscess drainage. This review emphasizes the expanding applications of ultrasound in camel medicine, particularly abdominal and thoracic interventions including abdominocentesis, thoracocentesis, portocentesis, and organ-specific biopsies. Ultrasound also aids in differentiating ascitic fluid types, improving diagnosis of complex diseases like peritonitis and uroperitoneum. Despite these advantages, challenges persist, including the need for camel-specific equipment, operator expertise, and adaptation for field conditions. Future efforts should prioritize standardizing protocols, integrating AI and telemedicine, and enhancing training to broaden accessibility and reliability. Incorporating ultrasonography into routine camel veterinary care, tailored to their unique biology and management, promises to elevate animal health and productivity. This advancement is vital for supporting pastoral communities reliant on camels across arid regions, ultimately improving both animal welfare and livelihoods. For practitioners, the key takeaway is that adopting ultrasound-guided interventions can significantly improve diagnostic precision and therapeutic outcomes in camels, enhancing both clinical decision-making and overall herd health.
